# Lesion of the subiculum reduces the spread of amyloid beta pathology to interconnected brain regions in a mouse model of Alzheimer’s disease

**DOI:** 10.1186/2051-5960-2-17

**Published:** 2014-02-11

**Authors:** Sonia George, Annica Rönnbäck, Gunnar K Gouras, Géraldine H Petit, Fiona Grueninger, Bengt Winblad, Caroline Graff, Patrik Brundin

**Affiliations:** 1 Neuronal Survival Unit, Department of Experimental Medical Science, Wallenberg Neuroscience Center, Lund University, Lund, Sweden; 2 Center for Neurodegenerative Science, Van Andel Research Institute, Grand Rapids, Michigan, USA; 3 Alzheimer Disease Research Center, Karolinska Institutet, Stockholm, Sweden; 4 Experimental Dementia Research Unit, Department of Experimental Medical Science, Wallenberg Neuroscience Center, Lund University, Lund, Sweden; 5 CNS Discovery and Translation Pharma Research and Exploratory Development, F. Hoffmann-La Roche AG, Basel, Switzerland; 6 Department of Geriatric Medicine, Genetics unit, Karolinska University Hospital, Stockholm, Sweden

**Keywords:** Amyloid-β, Alzheimer’s disease, Subiculum, Transgenic APP arctic mice, Prion-like, Ibotenic acid

## Abstract

**Background:**

The progressive development of Alzheimer’s disease (AD) pathology follows a spatiotemporal pattern in the human brain. In a transgenic (Tg) mouse model of AD expressing amyloid precursor protein (APP) with the arctic (E693G) mutation, pathology spreads along anatomically connected structures. Amyloid-β (Aβ) pathology first appears in the subiculum and is later detected in interconnected brain regions, including the retrosplenial cortex. We investigated whether the spatiotemporal pattern of Aβ pathology in the Tg APP arctic mice to interconnected brain structures can be interrupted by destroying neurons using a neurotoxin and thereby disconnecting the neural circuitry.

**Results:**

We performed partial unilateral ibotenic acid lesions of the subiculum (first structure affected by Aβ pathology) in young Tg APParc mice, prior to the onset of pathology. We assessed Aβ/C99 pathology in mice aged up to 6 months after injecting ibotenate into the subiculum. Compared to the brains of intact Tg APP arctic mice, we observed significantly decreased Aβ/C99 pathology in the ipsilateral dorsal subiculum, CA1 region of the hippocampus and the retrosplenial cortex; regions connecting to and from the dorsal subiculum. By contrast, Aβ/C99 pathology was unchanged in the contralateral hippocampus in the mice with lesions.

**Conclusion:**

These results, obtained in an animal model of AD, support the notion that Aβ/C99 pathology is transmitted between interconnected neurons in AD.

## Background

Recent studies in transgenic (Tg) mice modeling Alzheimer’s disease (AD) indicate that injected aggregates of amyloid-β (Aβ) [1,2] and tau [3-6] can seed aggregation of homologous proteins. Subsequently, the misfolded protein pathology can spread via anatomic connections, presumably through a prion-like intercellular transfer [1,7-11]. This mechanism could explain the stereotypic pattern of spreading of amyloid and tauopathy in the AD brain suggested by Braak and others [12-18]*.* A Tg AD mouse model (TgAPParc) over-expressing the human amyloid precursor protein (APP) with the arctic mutation (E693G) develops Aβ-neuropathology in a stereotypic topological and temporal pattern. The pathology first appears in the subiculum and gradually spreads to interconnected limbic brain regions over 3–15 months [7,19]. Previous studies describe lesions of neuronal pathways preventing APP delivery and Aβ deposition, leading to the removal of preexisting Aβ deposits [8,20]. This suggests that the development of pathology can be modified by interrupting the neural circuitry acting as a conduit for the prion-like transmission of Aβ.

The aim of this study is to test the hypothesis that disease-associated proteins transfer between anatomical regions in the brain and promote gradual spreading of neuropathology in a unique mouse model of AD, recapitulating the spatial and temporal development of Aβ pathology *in vivo*. We injected the excitotoxin ibotenic acid unilaterally into the dorsal subiculum in 6-week old Tg APParc mice, i.e. an age when the first signs of Aβ pathology have not yet appeared, to examine if destruction of neurons not only connecting to but also from the subiculum alter the spread of Aβ. In mice with subiculum lesions we observed reduced Aβ pathology at 3 months of age, when intracellular Aβ is normally present in the hippocampus and cortex, and at 6 months of age when diffuse Aβ deposits are expected to develop in the dorsal subiculum, alveus and retrosplenial cortex. This study supports the transmissibility of Aβ pathology between interconnected brain regions in Tg APParc mice.

## Methods

### Animals

We established colonies of homozygous TgAPParc and wild type (WT) mice in the Department of Experimental Medicine, Lund University, from previously characterized mice [7,19]. Tg APParc mice express human APP (695 isoform) under the mouse Thy1.2 expression cassette. At six weeks of age, Tg mice do not exhibit pathology [7,19]. Mice were maintained on an ad libitum diet with a 12-h light/dark cycle. A total of four to five female mice per group were analyzed. Lund University Animal Ethics Committee approved all animal experimental protocols.

### Ibotenic injections

We injected homozygous TgAPParc and WT mice aged six weeks, under isoflurane anesthesia. Mice received partial unilateral intrasubicular lesions by injecting ibotenic acid to the dorsal subiculum (0.5 μL) using a Hamilton syringe (coordinates, AP: -3.52, ML: 2.5, DV 1.35 mm relative to bregma and dural surface). As a control, homozygous TgAPParc mice received injections of 0.5 μL PBS at the same coordinates.

### Immunohistochemistry and Microscopy

Homozygous TgAPParc and WT mice aged 3 months were killed by cervical dislocation and the brain rapidly removed and stored on dry ice and 30 μm thick free-floating sections were cut on a freezing microtome. Six month old mice were anesthetized with sodium pentobarbitone and perfused transcardially with 0.9% saline followed by 4% paraformaldehyde (PFA) in phosphate buffer. Brains were removed and post-fixed in PFA for 4 hours before placing them in 30% sucrose until sectioning. 30 μm thick free-floating sections were cut on a freezing microtome and 3 and 6 month tissue immunostained with a primary antibody recognizing Aβ and C-terminal fragment of APP (C99), but not full-length APP [13] (antibody 82E1, IBL, Japan) following formic acid antigen retrieval, on six month old tissue. Sections were also immunostained using an antibody recognizing C-terminal APP, antibody 369 [21]. C99 refers to the Aβ-containing C-terminal fragment of APP that is generated after β-secretase cleavage, prior to γ-cleavage, which results in the formation of Aβ. Antibody 369 detects the C-terminal part of APP but does not detect Aβ. Sections were counter stained with haemotoxylin. For the purpose of cell counting, sections were stained using the cresyl violet stain. Sections were analyzed with a conventional light microscope (Eclipse 80i microscope; Nikon).

### Stereology

Total cell number and the number of Aβ/C99 positive cells (defined as cells containing one or more 82E1 immunoreactive puncta) were evaluated in the dorsal subiculum, CA1 and RSG (see Additional file 1: Figure S1). The brain regions were outlined in sections 240 μm apart using a 10x objective and then analyzed using a 63x oil immersion objective. The number of cells was quantified using computer-assisted mapping and a cell quantification program (Stereo Investigator, MBF Bioscience, Williston, USA) coupled to a Zeiss Imager M2 microscope (Carl Zeiss Microimaging, Göttingen, Germany).

The following definitions and settings were used: the subiculum was defined as the area closest to the CA1 and retrosplenial granular cortex (RSG). Subicular cells were evaluated using a counting frame of 140 × 90 μm, and a sampling grid (x = 155, y = 155). To define the CA1 region, the CA1 pyramidal cell layer was examined throughout the hippocampus until the pyramidal cell layer no longer formed a continuous loop [22]. This corresponded to -2.92 mm post bregma as described in Long et al. [23]. Counts were taken at predetermined intervals (x = 101, y = 161), using a counting frame (30 × 30 μm = 900 μm^2^). The RSG was defined as the area closest to the dorsal subiculum. RSG cells were evaluated using a counting frame of 140 × 90 μm, and a sampling grid (x = 155, y = 155).

Analyses of the Aβ/C99 pathology at three and six months of age was performed using stereology as described above where positive cells were counted in both hemispheres and a difference in the levels in the ibotenate-injected vs. intact hemisphere was calculated as a percentage.

### Densitometry

To quantify Aβ/C99 levels in the dorsal subiculum in 6-month-old mice exhibiting diffuse Aβ pathology, five images separated by 240 μm were taken using a 10x objective and centering the camera over the dorsal subiculum. The optical density and area measurements were analyzed using the computer program Image J. Previous studies have shown that OD measurements reflect changes in protein expression [24]. An example of the pathology analyzed using densitometry is in Additional file 1: Figure S1C.

### Statistical analyses

Data are presented as mean ± standard error of the mean (S.E.M.). Differences between groups were examined using Mann–Whitney test or Kruskal-Wallis followed by a Dunn’s post-hoc test or using Spearman’s correlation (Prism, GraphPad, La Jolla, CA USA).

## Results

**Figure 1:**
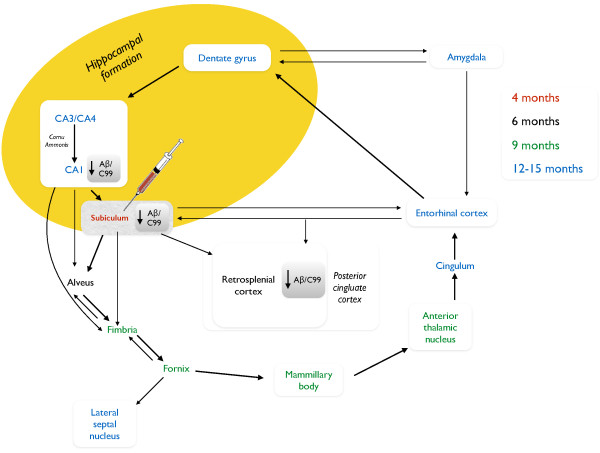
**Illustration of the progressive diffuse amyloid deposition in homozygous TgAPParc mice with altered pathology following subiculum lesion.** Anatomical connections between brain regions are marked by arrows. Colored text indicates the age when the first diffuse amyloid deposits are detected in the respective brain regions. Syringe marks the site of ibotenic acid lesion. Amyloid deposition affects functionally connected brain regions sequentially from the subiculum and brain structures receiving input from subiculum including the retrosplenial cortex, mammillary body, and thalamus. Decreased Aβ/C99 pathology was observed in the subiculum, CA1 and RSG (indicated). Schematic modified from Rönnback et al., 2012 [7], with permission.

The present study explores whether a lesion of the subiculum, achieved by injection of the excitotoxin ibotenic acid, can interrupt the spreading of Aβ pathology in Tg APParc mice which models the progressive spread of Aβ pathology over time (Figure 1). We applied the lesion in 6-week old Tg APParc mice that had not yet developed the first signs of Aβ pathology, and assessed in different brain regions connected to and from the lesion site for Aβ pathology in mice aged 3 and 6 months.

**Figure 2:**
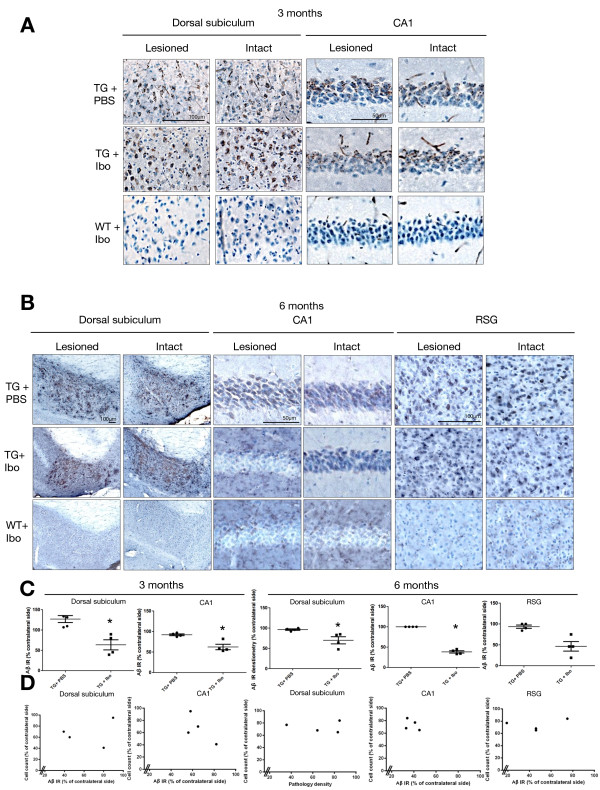
**Aβ/C99 immunoreactivity in dorsal subiculum, CA1 and RSG of Tg APParc mice that have undergone partial destruction of the subiculum. A**. Tg APParc mice aged 3 months comparing lesioned and intact hemispheres in the dorsal subiculum and CA1 of Tg mice injected with PBS (TG + PBS), Tg mice injected with ibotenic acid (TG + Ibo) and WT mice injected with ibotenic acid (WT + Ibo). We observed decreased Aβ pathology in both the dorsal subiculum and CA1 in Tg mice injected with ibotenic acid (Tg + Ibo). **B**. Aβ/C99 immunoreactivity in dorsal subiculum, CA1 and RSG of lesioned Tg APParc mice aged 6 months. Tg mice with ibotenic acid lesions have decreased Aβ/C99 immunoreactivity in the dorsal subiculum, CA1 and RSG. **C**. Quantification of the Aβ/C99 immunoreactivity comparing Aβ/C99 immunoreactivity (Aβ IR) in the lesioned hemisphere as a percentage of the contralateral side. Tg mice with ibotenic acid lesions have significantly decreased Aβ/C99 immunoreactivity in the damaged hemisphere in the dorsal subiculum and CA1 at ages 3 and 6 months. **D**. Plots representing cell count in the subiculum (percentage of contralateral side) vs. Aβ/C99 immunoreactivity in Tg + Ibo animals for the dorsal subiculum, CA1 and RSG of mice aged 3 and 6 months. Plots indicate a no correlation, r_s_ = 0.2, p > 0.05 (3 and 6 months dorsal subiculum, CA1 and RSG). Data expressed as means ± SEM. Asterisk denotes statistical significance (*p < 0.05).

At 3 months of age, we observed Aβ/C99 pathology as a cellular punctate signal in both the dorsal subiculum and in CA1 pyramidal neurons of Tg APParc mice (Figure 2A, Additional file 1: Figure S1A & B). In contrast, in WT animals, Aβ/C99 immunoreactivity was absent (Figure 2A). Importantly, in Tg APParc mice with lesions of the subiculum (Tg + Ibo), the Aβ pathology was significantly reduced in the dorsal subiculum and CA1 ipsilateral to the lesion compared to Tg APParc mice injected with PBS (Tg + PBS, *p < 0.05, Figure 2C).

We also assessed the levels of Aβ pathology in 6-month old mice. In the Tg APParc mice, Aβ pathology was evident as a diffuse signal in perikarya and neuropil in the dorsal subiculum and in the cytoplasm of CA1 pyramidal neurons and the RSG (Additional file 1: Figure S1C and D). Once again, there was no Aβ pathology detected with the Aβ/C99 antibody (82E1) in WT mice (Figure 2B). We performed densitometric analyses of the Aβ pathology at 6 months of age in the dorsal subiculum and stereological counts of Aβ/C99-immunoreactive cells in the CA1 and RSG. In Tg APParc mice that had been given ibotenate injections into the subiculum over 4 months earlier, the levels of Aβ/C99 pathology were significantly reduced in the dorsal subiculum and CA1 when compared to intact Tg APParc mice (*p < 0.05, Figure 2B and C). Regarding Aβ/C99-immunoreactive cells in the RSG, there was a non-significant trend for a decrease in mice with lesions (p = 0.0571, Figure 2B & C). The relationship between the percentage of cells remaining in the subiculum following the lesion (cell count% contralateral side) and Aβ/C99 pathology in subiculum, CA1 and RSG (% Aβ immunoreactivity) in Tg + Ibo mice were plotted (Figure 2D). The plots indicate that there is no significant correlation (r_s_ = 0.2, p > 0.05). Due to the small group size, strong conclusions cannot be drawn from the relationship between cell count and Aβ/C99 pathology.

The antibody 369 was used to evaluate the contribution of the C99 fragment of APP to the pathology following partial lesion to the dorsal subiculum in mice aged 6 months. This antibody detects APP outside of the Aβ region (Additional file 2: Figure S3). Immunostaining using the antibody 369 did not reflect the same changes as observed with the antibody 82E1, suggesting that the C99 fragment does not account for all the changes observed. It is more likely that Aβ is decreased and not the APP C-terminal fragments following the partial lesion. Nevertheless, we will continue to use the term Aβ/C99, since comparisons with different antibodies can be challenging and we cannot fully rule out a contribution of the C99 fragment of APP in the 82E1 labelling.

We then asked the question whether the significant decreases in Aβ/C99 pathology in the CA1 and RSG in mice could be due to cell death in distant brain regions following the injection of ibotenate into the subiculum. Therefore we performed stereological neuronal cell counts on cresyl violet stained tissue from mice aged 3 and 6 months and assessed cell loss not only in the dorsal subiculum, but also in the CA1 and RSG (Figure 1, Additional file 3: Figure S2A - C).

**Figure 3:**
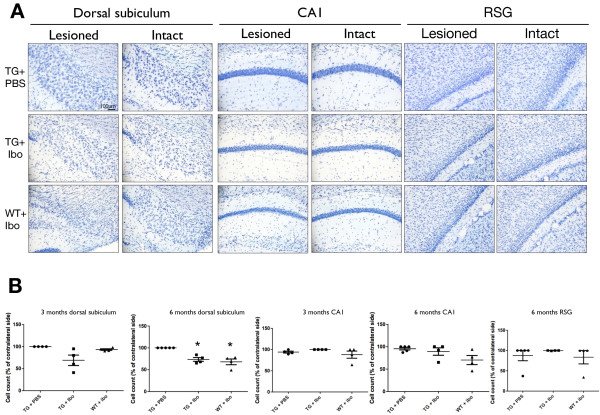
**Cell counts in the dorsal subiculum, CA1 and RSG of lesioned Tg APParc mice aged 3 and 6 months. A**. Representative images of cresyl violet stained tissue of Tg APParc mice aged 6 months comparing lesioned and intact hemispheres in the dorsal subiculum, CA1 and RSG of Tg mice injected with PBS (Tg + PBS), Tg mice injected with ibotenic acid (TG + Ibo) and WT mice injected with ibotenate (WT + Ibo). **B**. Quantification of cell counts comparing the percentage cell loss to the contralateral side. Data expressed as means ± SEM. Asterisk denotes statistical significance (*p < 0.05). CA1 and RSG (indicated).

At 3 months of age, the number of surviving neurons (large cell bodies in cresyl violet stained sections) in the dorsal subiculum varied greatly between mice. At 6 months, the number of surviving neuron-like cells in the dorsal subiculum was significantly reduced on the side of the lesion compared to the intact hemisphere (*p < 0.05, Figure 3B). Most importantly, the number of surviving cells in the CA1 and RSG regions was not lower on the side of the subiculum lesion compared to the intact hemispheres. This strongly supports the notion that cell loss in the CA1 and RSG does not explain the reduction in Aβ/C99 pathology (Figure 3B).

## Discussion

We show that a unilateral ibotenate-induced lesion of the dorsal subiculum in a Tg AD mouse model of progressive AD-related pathology, results in a decrease in Aβ immunoreactivity (the N-terminal of Aβ and the C99 fragment) in brain regions projecting to and receiving projections from this region. Thus in Tg APParc mice with excitotoxic lesions of the subiculum, we observed reduced Aβ staining in perikarya and neuropil in the dorsal subiculum and in the cytoplasm of CA1 pyramidal neurons and the RSG. The dorsal subiculum and CA1 send projections to the RSG [25,26]. The CA1 sends projections to the dorsal subiculum [22] but there is controversy over the possible projection from the dorsal subiculum to the CA1 [27]. We performed stereological cell counts, which revealed that, in our experimental paradigm, the decrease in pathology is not due to cell loss in the brain regions connected to the subiculum.

Progressive development of neuropathology, following stereotypic anatomical patterns, has been documented in various models of neurodegenerative diseases [28]. Regarding Aβ pathology, there is substantial experimental evidence that it can be triggered by intracerebral injections of, e.g., brain extracts containing Aβ and spread from the injection site in a prion-like manner [1,2,11,12,29]. Earlier studies in animal models of AD have demonstrated that Aβ can act as a “seed” and transmit Aβ pathology to unaffected brain regions. Thus, in young Tg AD mice that do not yet exhibit Aβ pathology, intracortical injections of Aβ-containing brain homogenates from AD patients or old transgenic mice can trigger the formation of Aβ plaques [1,8,14,30]. The pathology-inducing effects of the injected human AD brain extracts are both concentration- and time-dependent [1,2,11,29]. Recent work has also shown that it is sufficient with small amounts of Aβ present on a surgical instrument used for stereotactic surgery in AD mice to seed pathology [12]. Remarkably, even intraperitoneal injections of Aβ aggregates can seed Aβ pathology in the brain after several months [14]. Taken together, these studies have clearly shown the inducible nature of Aβ to act as a “seed” [1,12,14,16,18,30]. Not all of these earlier studies have described changes in Aβ pathology in brain regions both up and downstream of the site of an intracerebral injection of Aβ-containing extract. Interestingly, knife lesions of the perforant pathway have been reported to prevent APP delivery and Aβ deposition in the dentate gyrus of the hippocampus, and even result in a reduction in preexisting Aβ deposits in interconnected brain regions [8].

We have now extended these observations in a different transgenic AD model, i.e. Tg APParc mice that recapitulates the stereotypic development of AD pathology, and have used a different approach (excitotoxin-induced lesion) to destroy projecting neurons, assessing both up and downstream connecting brain regions. In Tg APParc mice and other APP transgenic mouse models, Aβ is deposited inside neurons prior to the extracellular space [7,8,19,20,31] and these mice develop diffuse plaques over time [7,19]. Following lesions of the subiculum, we observed reductions in both intracellular punctate/cytoplasmic Aβ signal (at 3 months of age) and extracellular Aβ/C99 in the subiculum (at 6 months, Figure 1C). The changes in the Aβ/C99 signal we observed is therefore likely due to reduced net transport of Aβ/C99 to and from the subiculum, possibly due to impaired axonal transport in remaining cells that are perturbed but not killed by the excitotoxin. APP and C-terminal fragments are transported within axons by fast anterograde axonal transport using kinesin-1 molecular motors and they accumulate in terminal fields [32-35]. A small population of APP is retrogradely transported [13,36]. APP can undergo further processing and full length APP and C-terminal fragments have been localized to neuritic vesicles [21,37]. A possible explanation for the reduction of Aβ pathology in CA1 and RSG following subiculum lesions in the Tg APParc mice could be the reduced transport and or delivery of APP and/or APP fragments (natively folded) from the subiculum to the CAI and RSG where APP is further processed and eventually aggregates [8,20,22]. The reduction of Aβ pathology we observed could also be the result of reduced transport of misfolded Aβ species that are released and cause prion-like seeding in interconnected regions [2,38]. If Aβ seeds spread from the subiculum in TgAPParc, further experiments would have to be performed to determine whether this occurs extracellularly, by diffusion from the subiculum along neuronal pathways, or intraaxonally. Future experiments could also explore if secondary effects play a role, e.g., potential changes in neuronal activity/connectivity influencing Aβ aggregation by altered expression of murine Aβ levels or related cofactors. Altered connectivity and therefore activity can in itself alter Aβ as a report indicates that removing whiskers in mice alters plaques and intraneuronal Aβ [39]. Furthermore, the possibility that loss of subicular cells (a source of human APP) could account for the reduction of pathology cannot be ruled out. However, it is assumed that the Aβ is transferred from the terminals, after it was first transferred (inter-cellularly) across the synapse to the cell bodies of the RSG. Removing the terminals (subiculum lesion) reduced transfer and hence the cell body staining for Aβ.

## Conclusions

Braak and colleagues [18,40] developed the hypothesis that in AD brains neuropathology progressively increases and spreads throughout the brain following a stereotypic pattern that appears to follow anatomical connections. This hypothesis was developed by examining post-mortem tissue and taking “snapshots” of disease progression. Our study provides support for the Braak hypothesis by demonstrating that the spreading of pathology from one brain region requires that the anatomical connections are intact [8,10,20,41]. Our study also strengthens the notion that the development of Aβ neuropathology in Tg APParc mice follows a spatiotemporal pattern that is dependent on neural connections. This suggests that this mouse model is highly relevant for future studies exploring novel interventions.

## Availability of supporting data

The data sets supporting the results of this article are included within the article.

## Abbreviations

AD: Alzheimer’s disease; Tg: Transgenic; APP: Amyloid precursor protein; Aβ: Amyloid-β; TgAPParc: Transgenic amyloid precursor protein arctic mice; WT: Wild type; PFA: Paraformaldehyde; C99: C-terminal fragment of amyloid precursor protein; RSG: Retrospenial granular cortex; Tg + Ibo: Transgenic with ibotenic injection; Tg + PBS: Transgenic with phosphate buffered solution injection; WT + Ibo: Wild type with ibotenic injection.

## Competing interests

The authors declare that they have no conflict of interest.

## Authors’ contributions

S.G. designed, managed and performed all the experiments. Analyzed the data and wrote the paper, A.R. assisted with experimental design, provided advice and input to the manuscript, G.K.G assisted with analyzing data, consultation and provided manuscript input, G.H.P helped design experiments and provided input to the manuscript, F.G. assisted with experiment design and provided manuscript input, B.W. consulted and provided manuscript input, C.G, contributed to the initiation of the project, conceived experiments, and consulted and P.B. contributed to the initiation of the project, conceived experiments, provided assistance with analysis and manuscript input. All authors read and approved the final manuscript.

## Supplementary Material

Additional file 1: Figure S1 Examples of positive Aβ/C99 staining in 3 month and 6 month old Tg APParc mice used for cell counting and densitometry analyses. A. Representative image of Aβ/C99 stained tissue of 3 month Tg APParc mice in the dorsal subiculum. B. Representative image of Aβ/C99 stained tissue of 3 month Tg APParc mice in the CA1. C. Representative image of Aβ/C99 stained tissue of 6 month Tg APParc mice in the dorsal subiculum. D. Representative image of Aβ/C99 stained tissue of 6 month Tg APParc mice in the CA1. E Representative image of Aβ/C99 stained tissue of 6 month Tg APParc mice in the RSG. Click here for file

Additional file 2: Figure S3 Example of dorsal subiculum C-terminal APP labeling in 6 month old Tg APParc following partial ibotenic acid lesion. A. Representative image of tissue from the lesioned dorsal subiculum from 6 month old Tg APParc mouse following partial lesion. B. Representative image of tissue from the intact dorsal subiculum from 6 month old Tg APParc mouse. Click here for file

Additional file 3: Figure S2 Example of cell counts in the dorsal subiculum of Tg APParc. A. Representative image of cresyl violet stained tissue of large dorsal subicular cells counted from Tg APParc mouse. B. Example of delineation of dorsal subiculum from Tg APParc mouse. C. Example of cell loss in of dorsal subiculum from a Tg APParc mouse with an ibotenate lesion. Click here for file
