# Correction: Lesion of the subiculum reduces the spread of amyloid beta pathology to interconnected brain regions in a mouse model of Alzheimer’s disease

**DOI:** 10.1186/s40478-023-01712-9

**Published:** 2024-02-14

**Authors:** Sonia George, Annica Rönnbäck, Gunnar K. Gouras, Géraldine H. Petit, Fiona Grueninger, Bengt Winblad, Caroline Graff, Patrik Brundin

**Affiliations:** 1 Neuronal Survival Unit, Department of Experimental Medical Science, Wallenberg Neuroscience Center, Lund University, Lund, Sweden; 2 Center for Neurodegenerative Science, Van Andel Research Institute, Grand Rapids, MI USA; 3 Alzheimer Disease Research Center, Karolinska Institutet, Stockholm, Sweden; 4 Experimental Dementia Research Unit, Department of Experimental Medical Science, Wallenberg Neuroscience Center, Lund University, Lund, Sweden; 5 CNS Discovery and Translation Pharma Research and Exploratory Development, F. Hoffmann-La Roche AG, Basel, Switzerland; 6 Department of Geriatric Medicine, Genetics Unit, Karolinska University Hospital, Stockholm, Sweden

**Correction: Acta Neuropathol Commun 2, 17 (2014)** 10.1186/2051-5960-2-17

Following publication of the original article [1], the authors identified an error in Fig. 2b. The correct figure and caption is given below.

Two panels in figure 2b were images captured from the same mouse by error and have been removed. Accessing the original data is not possible, as the files no longer exist due to the considerable time elapsed since publication, and therefore the images cannot be replaced. The original files were obtained in a laboratory in Sweden in 2013 and were relocated to the laboratory when it moved to the USA later the same year. Because this laboratory closed in early 2022 (before the authors became aware of the error), and the long time elapsed since publication of the paper, we have not been able to locate copies of the relevant original image files. The deleted images do not change the conclusions from the study.

The incorrect Fig. 2:
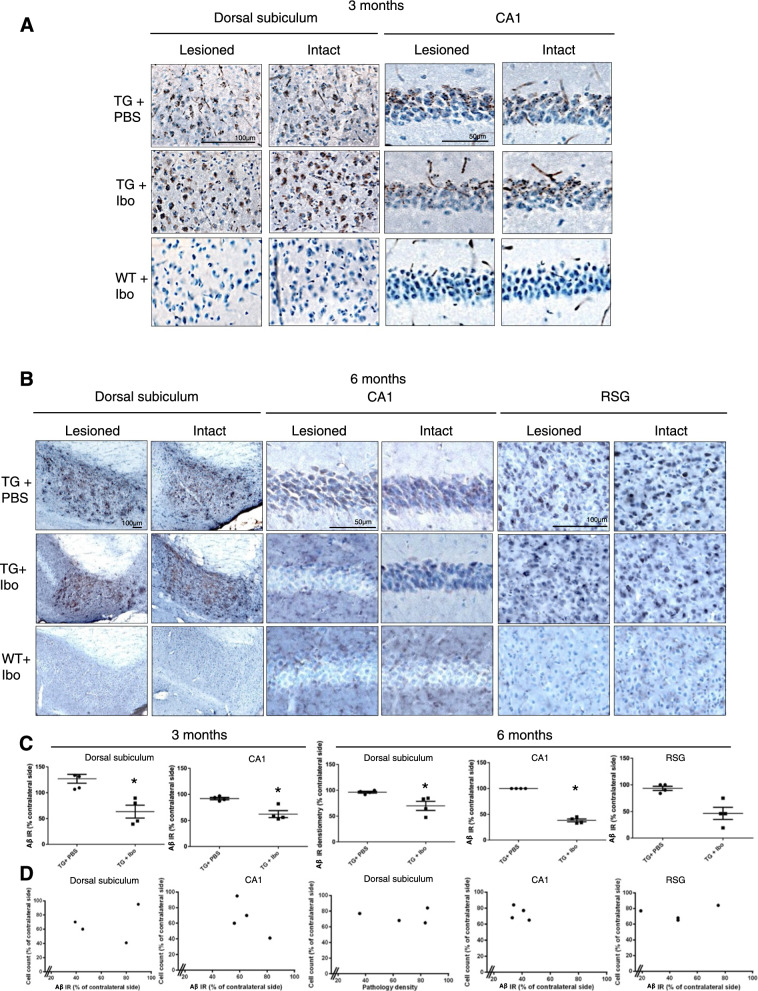


**Fig. 2** Aβ/C99 immunoreactivity in dorsal subiculum, CA1 and RSG of Tg APParc mice that have undergone partial destruction of the subiculum. **A** Tg APParc mice aged 3 months comparing lesioned and intact hemispheres in the dorsal subiculum and CA1 of Tg mice injected with PBS (TG + PBS), Tg mice injected with ibotenic acid (TG + Ibo) and WT mice injected with ibotenic acid (WT + Ibo). We observed decreased Aβ pathology in both the dorsal subiculum and CA1 in Tg mice injected with ibotenic acid (Tg + Ibo). **B** Aβ/C99 immunoreactivity in dorsal subiculum, CA1 and RSG of lesioned Tg APParc mice aged 6 months. Tg mice with ibotenic acid lesions have decreased Aβ/C99 immunoreactivity in the dorsal subiculum, CA1 and RSG. **C** Quantification of the Aβ/C99 immunoreactivity comparing Aβ/C99 immunoreactivity (Aβ IR) in the lesioned hemisphere as a percentage of the contralateral side. Tg mice with ibotenic acid lesions have significantly decreased Aβ/C99 immunoreactivity in the damaged hemisphere in the dorsal subiculum and CA1 at ages 3 and 6 months. **D** Plots representing cell count in the subiculum (percentage of contralateral side) vs. Aβ/C99 immunoreactivity in Tg + Ibo animals for the dorsal subiculum, CA1 and RSG of mice aged 3 and 6 months. Plots indicate a no correlation, rs = 0.2, p > 0.05 (3 and 6 months dorsal subiculum, CA1 and RSG). Data expressed as means ± SEM. Asterisk denotes statistical significance (*p < 0.05).

The correct Fig. 2:
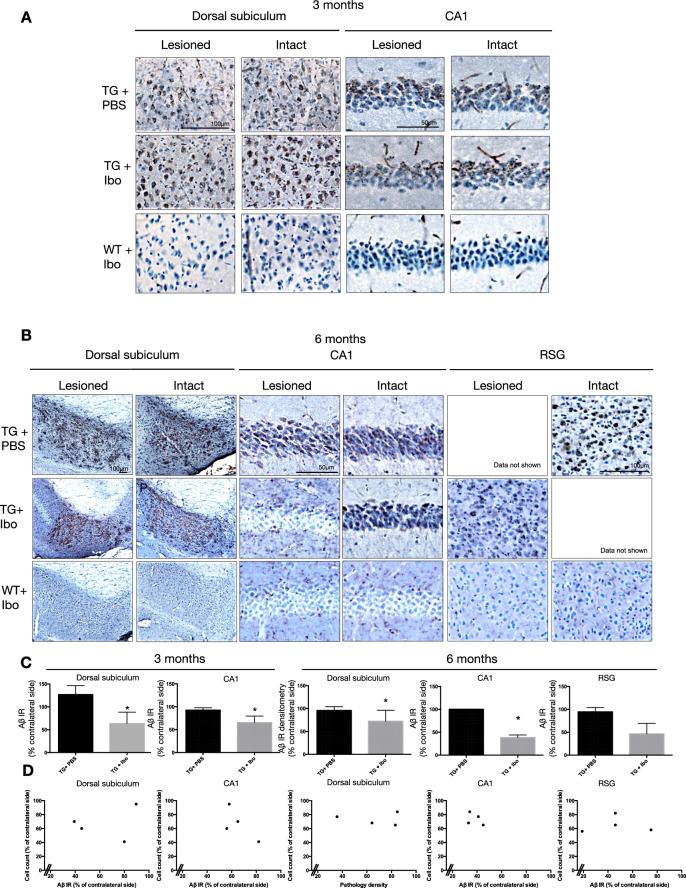


**Figure 2b**. Aβ/C99 immunoreactivity in dorsal subiculum, CA1 and RSG of Tg APParc mice that have undergone partial destruction of the subiculum. **A**. Tg APParc mice aged 3 months comparing lesioned and intact hemispheres in the dorsal subiculum and CA1 of Tg mice injected with PBS (TG + PBS), Tg mice injected with ibotenic acid (TG + Ibo) and WT mice injected with ibotenic acid (WT + Ibo). We observed decreased Aβ pathology in both the dorsal subiculum and CA1 in Tg mice injected with ibotenic acid (Tg + Ibo). **B**. Aβ/C99 immunoreactivity in dorsal subiculum, CA1 and RSG of lesioned Tg APParc mice aged 6 months. Tg mice with ibotenic acid lesions have decreased Aβ/C99 immunoreactivity in the dorsal subiculum, CA1 and RSG. Data not shown due to an error in the original publication, and we have now removed the erroneous images. **C**. Quantification of the Aβ/C99 immunoreactivity comparing Aβ/C99 immunoreactivity (Aβ IR) in the lesioned hemisphere as a percentage of the contralateral side. Tg mice with ibotenic acid lesions have significantly decreased Aβ/C99 immunoreactivity in the damaged hemisphere in the dorsal subiculum and CA1 at ages 3 and 6 months. **D**. Plots representing cell count in the subiculum (percentage of contralateral side) vs. Aβ/C99 immunoreactivity in Tg + Ibo animals for the dorsal subiculum, CA1 and RSG of mice aged 3 and 6 months. Plots indicate a no correlation, rs = 0.2, p > 0.05 (3 and 6 months dorsal subiculum, CA1 and RSG). Data expressed as means ± SEM. Asterisk denotes statistical significance (*p < 0.05).

Figure 2b has been updated above and the original article [1] has been corrected.
