# Supplementary figures and images for: Lesion of the subiculum reduces the spread of amyloid beta pathology to interconnected brain regions in a mouse model of Alzheimer’s disease
Source: Acta Neuropathol Commun. 2014 Feb 11;2:17. doi: 10.1186/2051-5960-2-17 (PMC3932948; doi:10.1186/2051-5960-2-17)

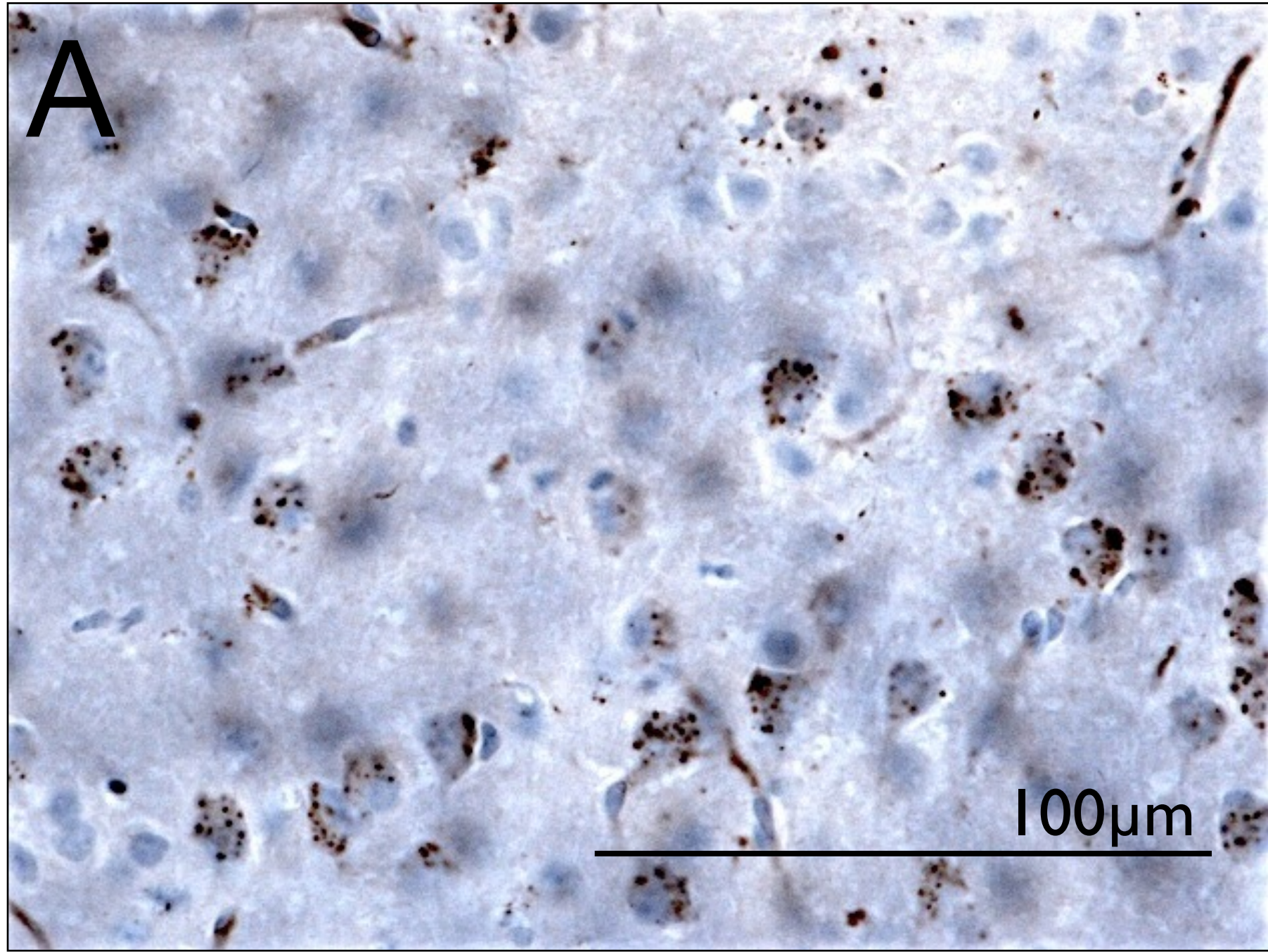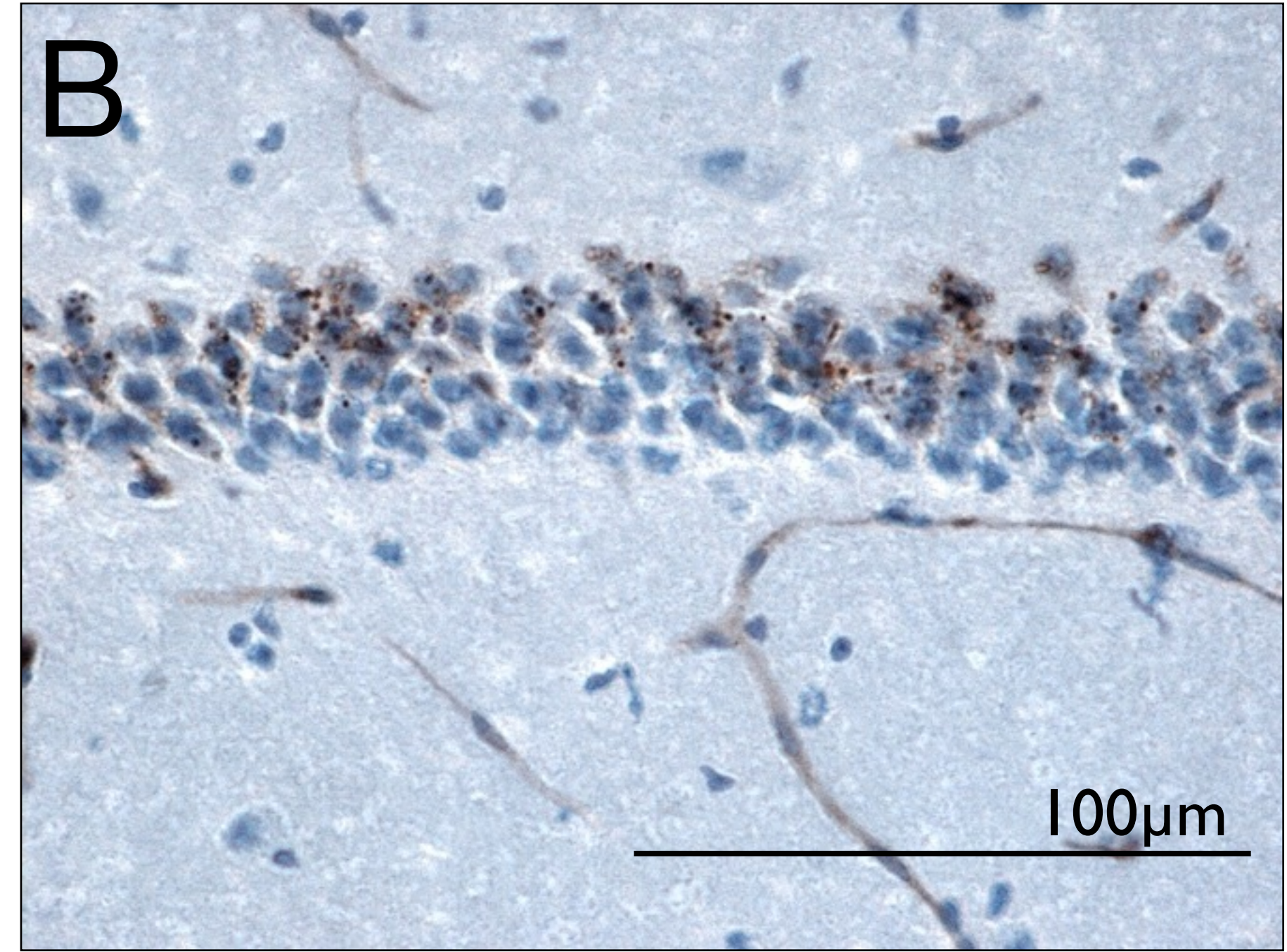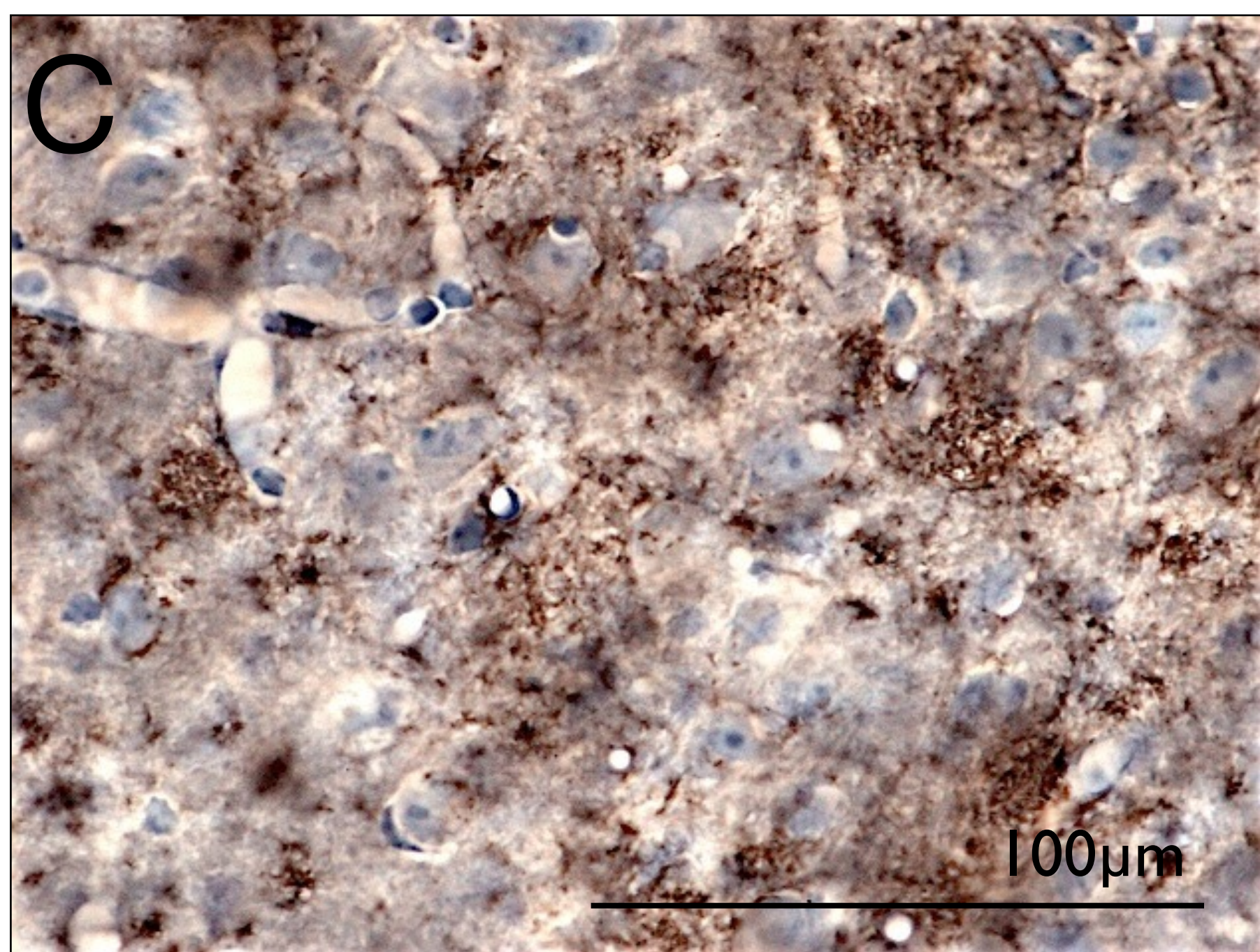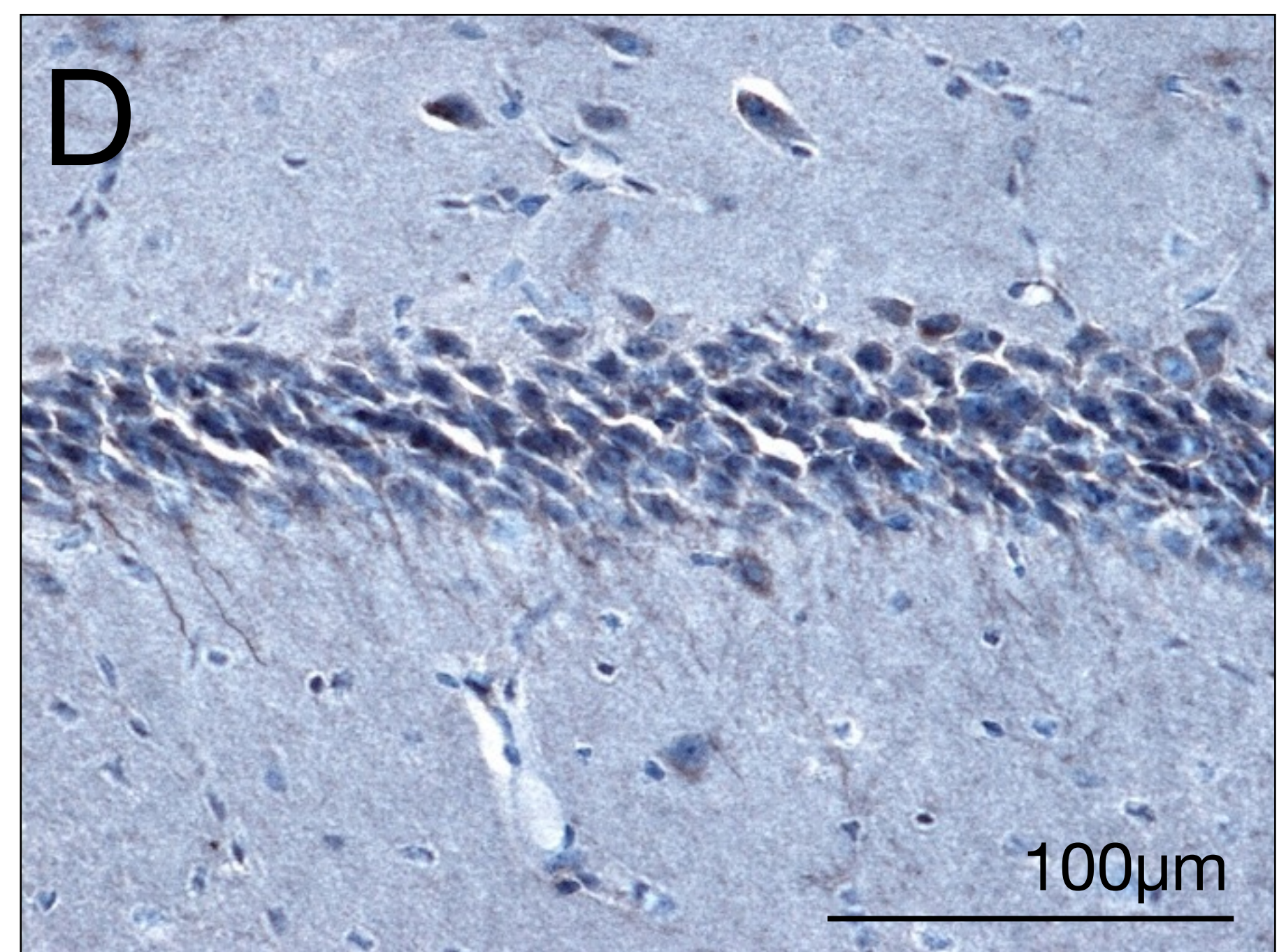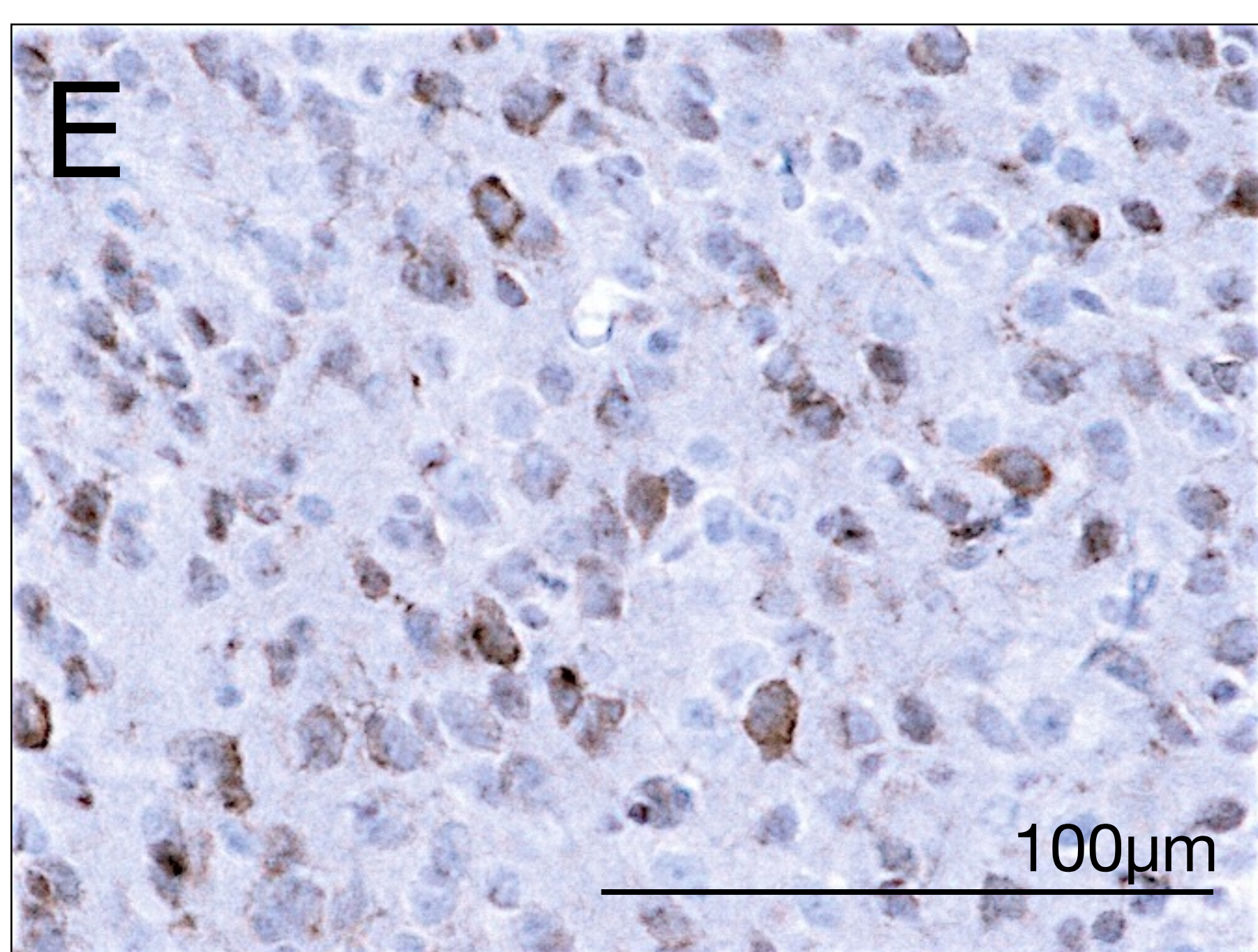

Supplement: Additional file 1: Figure S1 — Examples of positive Aβ/C99 staining in 3 month and 6 month old Tg APParc mice used for cell counting and densitometry analyses. A. Representative image of Aβ/C99 stained tissue of 3 month Tg APParc mice in the dorsal subiculum. B. Representative image of Aβ/C99 stained tissue of 3 month Tg APParc mice in the CA1. C. Representative image of Aβ/C99 stained tissue of 6 month Tg APParc mice in the dorsal subiculum. D. Representative image of Aβ/C99 stained tissue of 6 month Tg APParc mice in the CA1. E Representative image of Aβ/C99 stained tissue of 6 month Tg APParc mice in the RSG. [file 2051-5960-2-17-S1.pdf]

6 months  
Dorsal subiculum

Lesioned

Intact

TG + Ibo

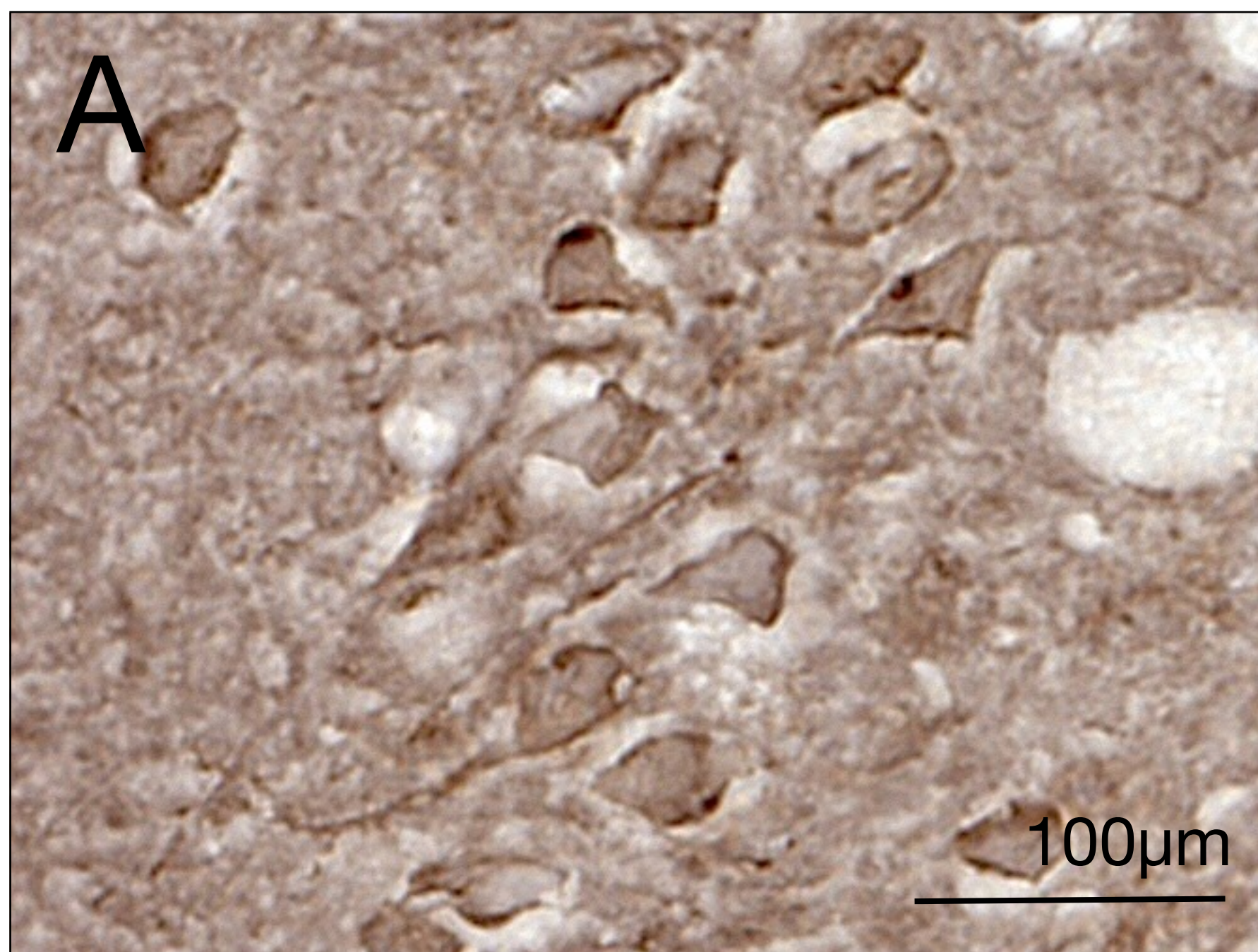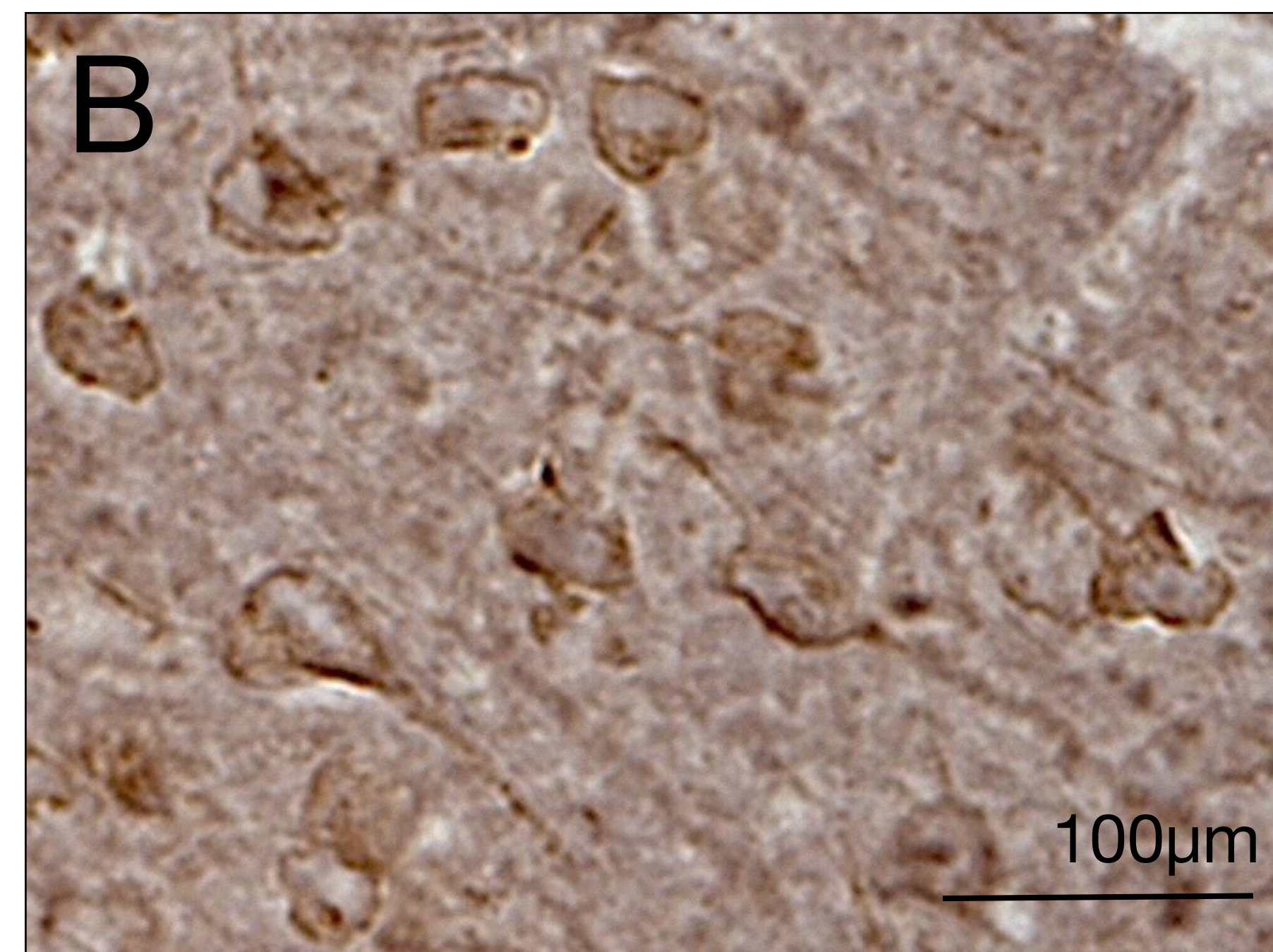

Supplement: Additional file 2: Figure S3 — Example of dorsal subiculum C-terminal APP labeling in 6 month old Tg APParc following partial ibotenic acid lesion. A. Representative image of tissue from the lesioned dorsal subiculum from 6 month old Tg APParc mouse following partial lesion. B. Representative image of tissue from the intact dorsal subiculum from 6 month old Tg APParc mouse. [file 2051-5960-2-17-S2.pdf]

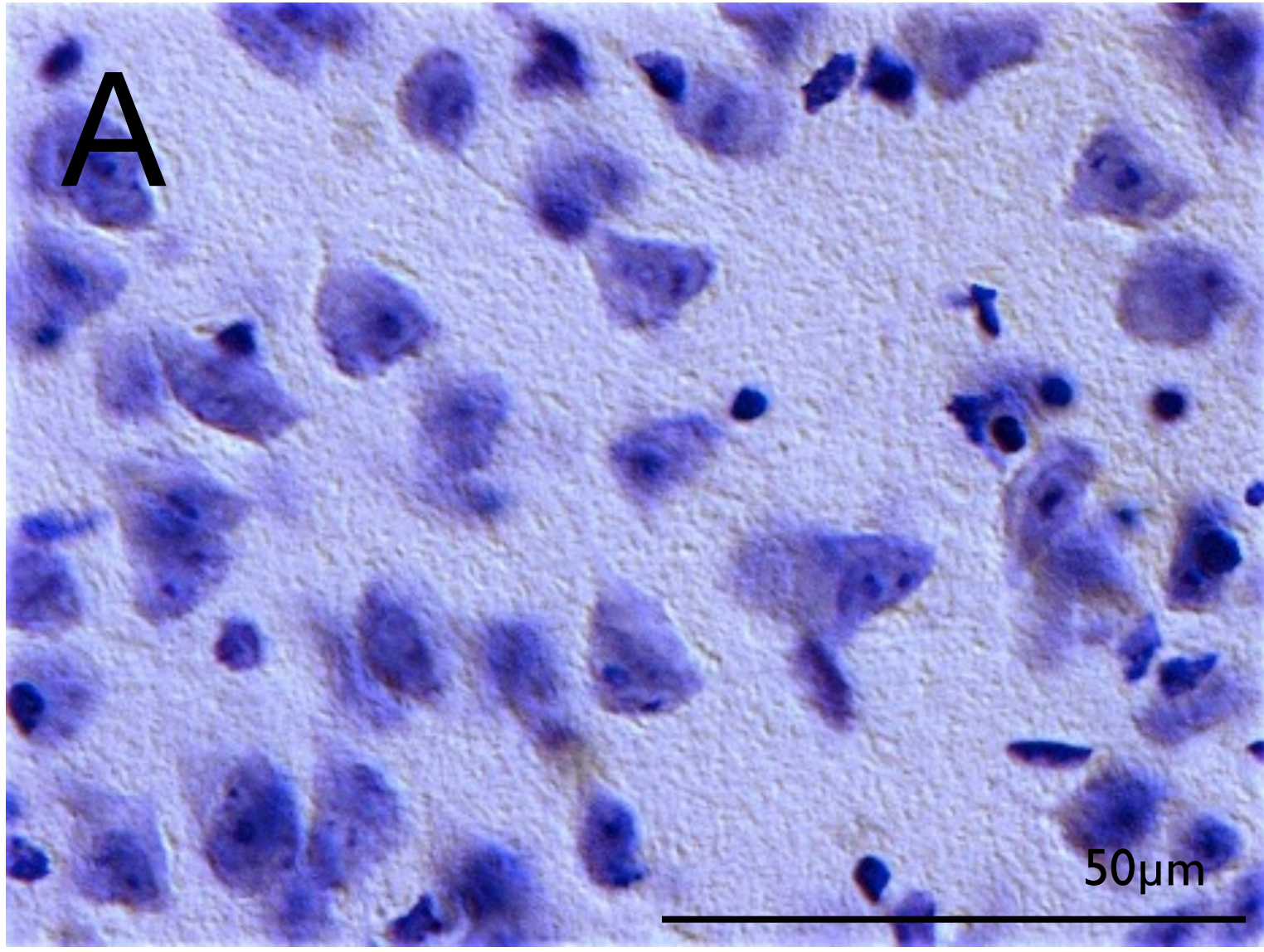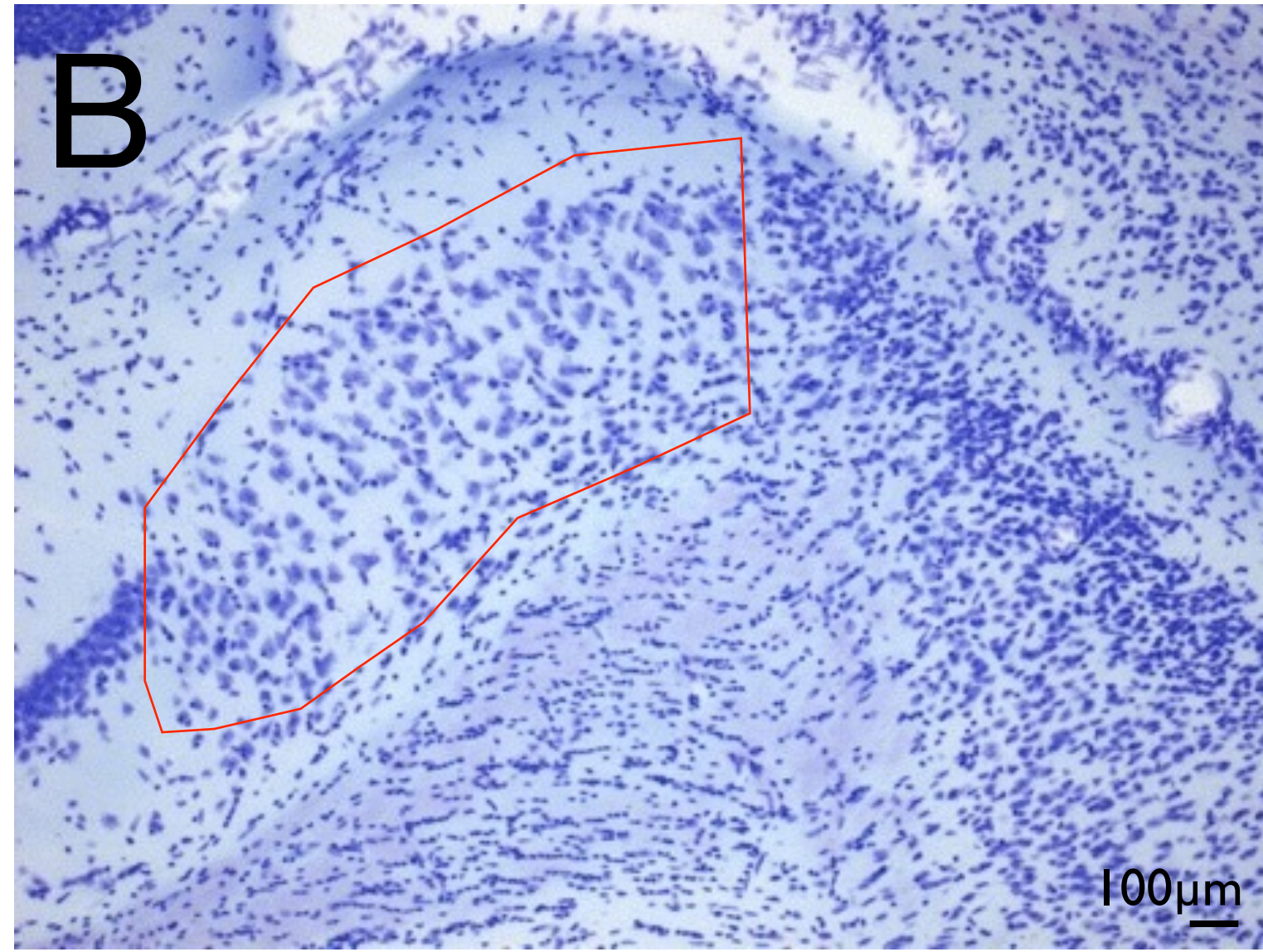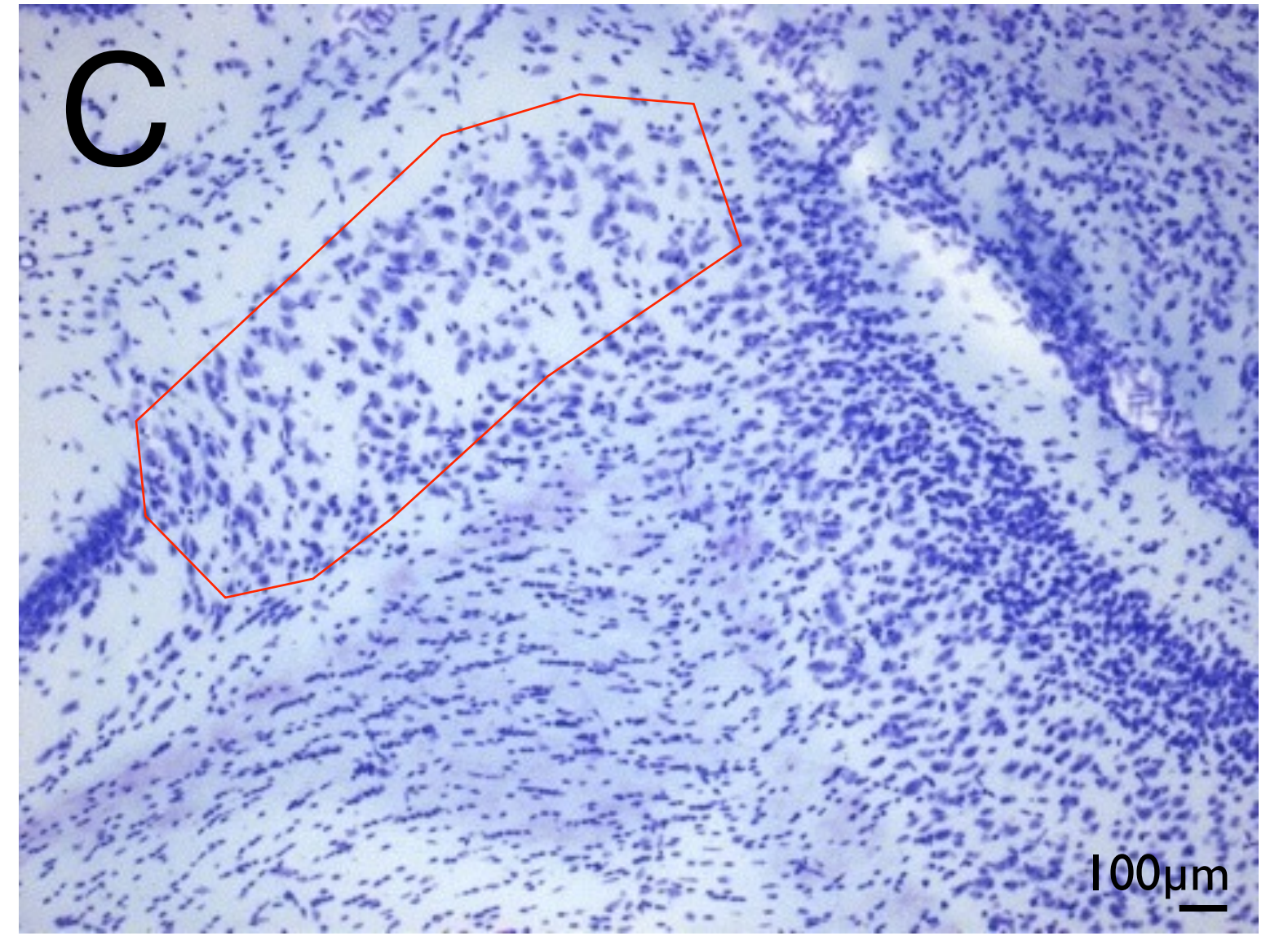

Supplement: Additional file 3: Figure S2 — Example of cell counts in the dorsal subiculum of Tg APParc. A. Representative image of cresyl violet stained tissue of large dorsal subicular cells counted from Tg APParc mouse. B. Example of delineation of dorsal subiculum from Tg APParc mouse. C. Example of cell loss in of dorsal subiculum from a Tg APParc mouse with an ibotenate lesion. [file 2051-5960-2-17-S3.pdf]
